# Review of anthrax: A disease of farm animals

**DOI:** 10.5455/javar.2022.i599

**Published:** 2022-06-30

**Authors:** Md. Emtiaj Alam, Md. Mostofa Kamal, Moizur Rahman, Aurangazeb Kabir, Md. Shafiqul Islam, Jayedul Hassan

**Affiliations:** 1 Department of Veterinary and Animal Sciences, Faculty of Veterinary and Animal Sciences, University of Rajshahi, Rajshahi, Bangladesh; 2 Department of Microbiology and Hygiene, Faculty of Veterinary Science, Bangladesh Agricultural University, Mymensingh, Bangladesh

**Keywords:** Anthrax, disease, farm animals, review

## Abstract

Anthrax is a rapidly fatal infectious disease affecting herbivores and people. In the farm animals, cattle and sheep are more susceptible, followed by goats and horses, while dwarf pigs and Algerian sheep are relatively resistant. *Bacillus anthracis*, the causative agent of anthrax, produces spores and persists for decades in the soil, initiating an outbreak through a favorable climate shift. Anthrax is enzootic in many Asian and African countries, and is reported in Australia, some parts of Europe, and America. The clinical courses of this disease in animals are peracute, acute, subacute, and chronic forms. In severely infected cases, the animals are dead without premonitory clinical signs. The blood may fail to clot and can be found in the mouth, nostrils, and anus in the animals that die from anthrax. This bacterium is susceptible to many antibiotics, yet only penicillin and oxytetracycline have the most effective under field conditions. When an outbreak occurs in a defined area, it is necessary to take early steps to break the infection cycle by maintaining strict biosecurity and vaccinating uninfected animals. This disease is still a challenge to farm animal production in many countries. This review intends to give a fair knowledge of the etiology, epidemiology, pathogenesis, clinical presentation, diagnosis, treatment, and control of this disease.

## Introduction

Anthrax is a zoonotic disease caused by the bacterium *Bacillus anthracis* [1]. This organism is an aerobic, or facultatively anaerobic, capsulated bacterium that produces spores upon exposure to the environment through different body fluids of a dead carcass, enabling long survival [2–6]. The vegetative cells do not last long outside the host body without sporulation. An unopened carcass over 72 h inhibits sporulation of the vegetative cells, resulting in the end of their life cycle [4,5,7]. Once sporulated, they are resistant to the hostile environment, including heat, cold, desiccation, chemical disinfection, salting hides, pH, and irradiation [6,8].

This disease affects farm animals and wildlife; occasionally, an outbreak occurs in humans and is distributed globally [2]. Among the farm animals, particularly cattle, sheep are more infected than goats and horses, and the dwarf pig and Algerian sheep species are relatively resistant [9].

Asian and African countries are considered the major reservoirs of anthrax [10,11]; however, this disease has been reported in the United States of America (USA) [12], Australia [13], Sweden [14], Italy [2], and many locations in Europe. In current years, anthrax of animals has occurred repeatedly in some parts of Bangladesh, especially the Sirajganj and Pabna districts [15]. The outbreak of this disease in Bangladesh indicates its pattern shift from sporadicity to endemicity in this country [15,16]. In Bangladesh, it has been reported in cattle, goats, buffaloes, elephants, and humans [17,18].

In animals, the clinical course of this disease occurs in peracute, acute, subacute, and chronic forms. In severely infected cases, animals may die within 48–72 h, and blood may fail to clot and exudes from the nose, mouth, and anus [19,20]. Strict biosecurity includes the supply of properly cleaned grasses, purchasing foods from reliable sources, using disinfectant (1% active sodium hypochlorite/bleach in water)containing footbath at the entrance,, hand washing with antimicrobial or non-antimicrobial soap and water, and using gloves while handling animals. Regular vaccination aids in controlling the disease in susceptible animals [21,22]. The different reports related to anthrax are available in various parts of the world. However, unified information like epidemiological, pathological, clinical, diagnostic and preventive aspects of this disease in farm animals is still lacking.

In addition, from the ancient period, anthrax has caused huge production loss in both domestic and wild animals, but after the discovery of the vaccine, its incidence was much reduced. In the last three decades, it has become sporadic in most regions where regular vaccination programs are implemented for livestock animals. It has zoonotic importance and potential as a bioweapon that everyone is concerned about [23].

This review aims to give a clear idea of the etiology, epidemiology, clinical presentation, pathology, diagnosis, treatment, and control of anthrax in farm animals and to further overcome the limitations described above.

## Etiology

Anthrax is caused by *B. anthracis*, a rod-shaped, Gram-positive (gm+), immobile, capsulated, aerobic or facultatively anaerobic, spore-forming bacterium, belonging to the family Bacillacae, order Bacillales, and genus *Bacillus* [2]. The spores are more vigorous than the vegetative form of this bacterium [24]. Soon after shading from the host and exposure to free oxygen, this bacterium sporulates. An environmental temperature between 53 and 107°F (12°C–42°C) facilitates sporulation of this bacterium, while sporulation of this *Bacillus* does not occur at temperatures below 48–53°F (9°C–12°C) [9,20]. When suitable conditions are favorable, the spores spark outbreaks of the disease [24], and the spores return to the vegetative form after the host animal, or human, is infected [9,20].

There are 89 known strains of *B. anthracis*. The most well-known strains of *B. anthracis* are Ames, Sterne, and Vollum. Virulence of this bacterium was associated with two plasmids (pX01 and pX02) that carry the genes coding for toxin and capsule synthesis, respectively. Although Ames 0581, which contains both plasmids, is considered the “gold standard” to compare the potency with others, Anthrax 836 is the most virulent strain described yet. In addition to their virulence, the strains have different evolutionary histories or origins. The strain 836 was discovered in Kirov in 1953 and weaponized by the Union of Soviet Socialist Republics (USSR) [25]. On the other hand, the Ames strain was isolated first in a dead cow in 1981 in Texas, USA [26], and the Sterne strain was found in Canada, an avirulent, toxigenic strain generally used in vaccine production [27]. The Vollum was isolated first in a cow in Oxford, England, and it was used for bioweapon trials in 1942 on Gruinard Island [28].

## Epidemiology

### Occurrence

The disease anthrax has been well known since ancient times (700 BC). As far as known, this disease has been thought to have originated in sub-Saharan Africa, especially Egypt and Mesopotamia. However, anthrax has been evidenced in ancient Greece and Rome through famous writings like Iliad by Homer at around 700 BC and poems by Virgil (who lived from 70 to 19 BC) and thought to have a worldwide distribution. Anthrax (cutaneous type) was clinically first described by Maret in 1752 and Fournier in 1769, and before their clinical representation, anthrax had only been reported historically. In 1877, Robert Koch carried out various experiments on anthrax bacteria and developed the famous Koch postulates, which mentioned a causal relationship between the disease and the specific microorganisms. In the late 19th and early 20th century, anthrax was reported to cause the highest case fatality in domestic and wild animals [9,23]. In 1923, an outbreak of this disease was estimated to cause the death of 30,000–60,000 animals in South Africa [29].

Anthrax is reported sporadically in North America, Western Europe, and Australia. Enzootic occurrence of this disease is documented in Greece, Spain, Southern Italy, Turkey, and Albania; however, this disease is absent in northern and central Europe. Latin American countries, including Mexico, Bolivia, and Peru, have contracted this disease with the enzootic occurrence in Haiti. This disease is found endemically in some countries of Asia, including eastern India, South Korea, the Philippines, Mongolia, and the mountainous region of western China [2,9]. African countries are suffering from outbreaks of this disease every summer with devastation in years of heavy rainfall [2,9].

In Bangladesh, periodic outbreaks of anthrax in both animals and humans were reported between 1949 and 2017. 450 animal and 725 human cases were reported in Bangladesh from 1980 to October 2010 [15]. Recently, the disease has changed, and enzootic outbreaks are reported yearly in some districts, especially Pabna and Sirajganj regions in Bangladesh.

Nowadays, it is receiving increased attention for use as a potential agent of bioterrorism. A historical bioterrorist attack was documented in the USA in the fall of 2001, where 30,000 people were contaminated with anthrax spores sent through post mail leading to 5 deaths and 17 others left infected [9,30].

### Infection source

Diverse hosts and environmental sources of anthrax have been described, including the soil, fodder, bone meal, infected excreta, blood, and other infected animal discharges. Most frequently, the initial source had come when the soils of old anthrax graves were disrupted. The bacilli spreads over the area through contaminated streams, insects, and fecal contamination of infected birds and feral animals, including cats, dogs, and other carnivores [9]. The infection is introduced into a new area by concentrates, forage, or contaminated animal products, like bone meal, hides, hair, wool, fertilizers, etc. [2,9].

### Transmission

**Figure 1.**
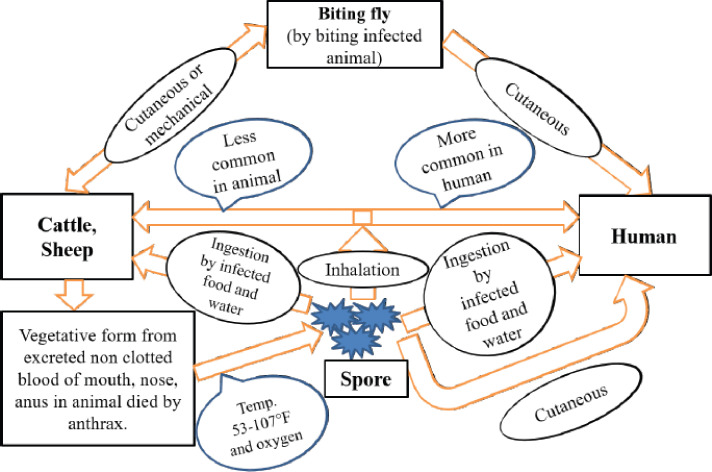
The transmission cycle of anthrax.

The bacilli enter the body via ingestion, inhalation, or penetration through disrupted skin. Figure 1 shows the transmission of anthrax in cattle, sheep, and humans. In most animal cases, ingestion of concentrate, forage, or water contaminated with anthrax bacilli is the principal route of infection. Any wound on the mucous covering of the digestive tract facilitates the entrance of the bacilli into the system. The inhalation infection is of minor importance in animals, although this infection type (by contaminated dust) is always considered [9,20]. Exposure to contaminated hair, wool, hides, and skins was the common route to inhalational and cutaneous types of anthrax in the personnel working in the related industries [9,20].

The biting (Stomoxys or Tabanus species) and nonbiting (blowfly and chrysoma) flies have been involved in several anthrax outbreaks in livestock and wildlife in India, Africa, and the USA [1]. This type of transmission is mechanical and, after infection, results in edema that progresses to the formation of painless black sores on the skin [9,31].

## Risk Factors

### Host risk factors

All vertebrates are prone to anthrax [9], but cattle have been infected more frequently than the other species [32]. Cattle are infected more than sheep and goats because cattle pull pasture out of the ground with roots, whereas sheep and goats bite plants off the ground level or browse on shrubs [32]. As a result, cattle can ingest high doses of bacilli from contaminated soil compared to other herbivores. The disease occurs less frequently in horses and goats than in cattle and sheep.

The pigs, dogs, and cats are relatively resistant to this disease. This disease is almost fatal in farm animals, except for pigs. However, like other farm animals, the case fatality rate is high in pigs. The Algerian sheep and dwarf pigs are resistant to anthrax. In the case of the dwarf pig, the anthrax spores remain ungerminated in tissues. All spores are completely clear from the tissues of the dwarf pig by 48 h. It is an inherited ability of the dwarf pig to prevent spore germination [9].

### Pathogen risk factors

The capsule and toxin complex encoded by two distinct plasmids pXO2 and PXO1, respectively, are the most important virulence factors in pathogenic strains of *B. anthracis*. The toxin complex comprises three proteins that are protective antigen (PA), edema factor (EF), and lethal factor (LF). The bacilli are inherently dependent on the capsule to establish in the host tissues. It also helps the *Bacillus* to evade the host immune system; by protecting the surface antigen of the *Bacillus*, and exposure to host antibodies. When the variants of bacilli loose the capsule, they also loose their virulence [33]. The anthrax toxin complex (PA, EF, and LF) acts synergistically. The previous study showed that the combined effect of three proteins resulted in more toxicity than each toxin effect separately [34]. PA is necessary for binding specific target cells and introducing EF and LF into the target cells. At low cytoplasmic pH, the EF generates a cAMP gradient that destroys the cells [35]. It also causes extracellular edema by losing chloride ions and water from the cells. It also impaired the phagocytic activity of neutrophils [36] and increased the motility of infected macrophages [31]. The LF inactivates the protein kinases in the cytoplasm of cells resulting in cell apoptosis [37]. It also enhances the tumor necrosis factor (TNF) and interleukin-1 (IL-1) inside the macrophages leading to the cell lysis and release of inflammatory mediators, resulting in cell apoptosis [36].

### Environmental risk factors

The anthrax outbreak was reported after significant climate changes like heavy rain after a prolonged drought, dry summer months after prolonged rain, late in the dry season after a prolonged drought, or warm weather [9,38]. The previous studies suggest that the outbreaks are related to the neutral or alkaline soil rich in calcium and nitrogen and temperature above 59.9°F (15.5°C) [39–41]. Surprisingly, environmental factors that influence *Bacillus* cell development also significantly impact sporulation. To the best of our knowledge, there is still no proof that *Bacillus* spores can develop outside the range of the temperature, pH, and water activity (*a*_w_) that allow growth. Sporulation yield is typically highest at optimal growth temperature, pH, or *a*_w_, and declines as temperature, pH, or *a*_w_ deviate from optimal, lengthening the sporulation process [42–47]. For example, in standard nutrition broth (optimal conditions), *Bacillus* takes 3 days to complete growth and sporulation at 37°C, pH 8.0, and high *a*_w_. It increases to 10 days at 45°C, 14 days at 19°C, 20 days at pH 6.0 or 10.0, and 17 days at *a*_w_ = 0.950 [46].

The sporulation of *B. anthracis* rapidly occurs in the environment when the environmental temperature is over 53°F (12°C) [48]. On the contrary, spore production was also seen at 10°C and 7°C but with much lower ability. Inhibition of *Bacillus* sporulation by more salinity (about 7% NaCl) happens at an early stage due to impaired activity of the response regulator Spo0A controlling entry into sporulation and the alternate sigma factor σH [49]. The number of spores generated (and cell growth) was smaller under the oxygen limitation than under aerobiosis, indicating that oxygen concentration affects *Bacillus* sporulation yields [50–52].

The anthrax spore has a high surface hydrophobicity; that is why the spores are clumpy, concentrated, and remain in the standing water. The spore has a further concentration on the soil surfaces after the water evaporates. These relationships between anthrax *Bacillus* and the climate may help predict the anthrax years [9,38].

## Economic Importance

Vaccination of susceptible animals in enzootic areas of the developing countries has decreased the disease prevalence to a negligible proportion nationally. However, heavy losses may still occur in individual herds. The loss happens as a result of animal mortality, milk withholding in diseased dairy herds, and for a period following immunization [9].

### Pathogenesis

Infection may occur through ingestion (from defect or any wound of the digestive tract), skin abrasions or skin lesions (from biting or nonbiting flies), and spore inhalation. Outside a host, the mature spores of *B. anthracis* are dormant. Upon entry into a host, the spore germination into mature *B. anthracis* occurs in the macrophages at the initial site of infection. In the case of inhalational anthrax, the germination occurs later upon arrival at the local lymph node. After spore uptake into phagolysosomes by tissue macrophages, the bacilli are transported via lymphatic channels to local and regional lymph nodes. Final germination occurs in the lymph nodes draining the primary site of infection; mature bacilli escape from macrophages and multiply systemically. Bacilli spread through the circulatory system, causing septicemia and infection of other internal organs of the host, and cause damage [53,54].

Animals and humans can be exposed to anthrax in three or four ways [55,56]. A previous study showed how the toxins of bacilli enter the cells and other pathogenic mechanisms of the bacilli [55].

Some factors contribute to the pathogenesis of *B. anthracis*. They are PA, EF, and LF, which combine to form two toxins: edema toxin (ET) and lethal toxin (LT). ET reduces the neutrophil function *in vivo* and imbalances water homeostasis leading to edema. LT causes delivery of IL-1β factors and TNF-α, which are believed to link the septic shock and sudden death in severe anthrax infection [57]. The capsule of *B. anthracis* inhibits phagocytosis. The full virulence of bacilli needs both the toxin components and an antiphagocytic capsule [55,58].

The severity of infection mostly depends on the total amount of toxin produced, the antiphagocytic capsule quality, the host susceptibility [9], and the high concentration of bacteria infecting the host [59]. The toxins act on the blood vessels’ endothelial lining, leading to breakage and bleeding. LT of *B. anthracis* directly inhibits whole-blood clotting, platelet aggregation, surface P-selectin expression, and platelet–endothelial cell interactions [60]. That is why the blood does not clot and may be present around the mouth, nose, and anus [20].

## Clinical Presentation

It is difficult to determine the incubation period after field infection, but it is usually 3–7 days following oral challenge [61]. In naturally infected cattle, the incubation period is 1–14 days or more [20]. The clinical course of anthrax in animals may be peracute, acute, subacute, and chronic. When an anthrax outbreak starts, the peracute form is most often seen in cattle, sheep, and goats. The acute and subacute conditions most often are found in cattle, sheep, and horses. The chronic form of anthrax is common in swine and reported in cattle, horses, dogs, and cats [19].

### Cattle, sheep, and goats

In the case of peracute anthrax for cattle, sheep, and goats, the clinical signs are fever, staggering, excitation/somnolence, recumbence, spasms, trembling, and dyspnea [19,62], and the infected animal will die within 48–72 h. The disease progression is rapid, and often animals are found dead without premonitory clinical signs. The dying animals are usually found bloated without rigor mortis or incomplete rigor mortis, and the absence of clotting of the blood is the most prominent characteristic of anthrax [1,20,63]. The blood may fail to clot, and blood discharges can occur from the nostrils, eyes, mouth, and anus after death [64].

The acute form may manifest in high fever (up to 107°F/42°C), excitement, the mucosa congested and hemorrhagic, tachycardia, labored breathing with terminal convulsions, and death [62]. Abortion, reduction of milk production, and discolored milk (blood-tinged or deep yellow) have been reported in dairy cattle [62]. Generally, the alimentary tract involvement occurs and is characterized by dysentery and diarrhea.

In the case of chronic anthrax, the characteristic clinical sign is swelling and edema in the subcutaneous area, usually seen in the brisket, shoulder, thorax, perineum, and flank [19]. Alimentary tract involvement is generally characterized by diarrhea and dysentery [9]. Localized tongue edema and edematous lesions in the throat, sternum, perineum, and flanks are also possible.

### Horses

In general, anthrax in horses is acute, and the clinical signs varying with the mode of infection. When infection occurs by ingestion, the clinical sign includes fever, labored breathing, colic, dysentery, and bloody discharge from the nose and anus [1,63]. The death of animals usually occurs within 48–96 h. When *B. anthracis* is introduced through an insect bite, it results in hot, painful, edematous, subcutaneous swelling at the bite site, followed by swelling that appears in the neck, sternum, ventral abdomen, prepuce, and mammary gland [9].

### Swine

Anthrax may develop in pigs in acute septicemia characterized by sudden death, oropharyngitis, or mild chronic form. In the case of oropharyngeal anthrax, the characteristic clinical sign is rapidly progressive swelling in the throat. The swellings are hot without pain and may cause an obstruction, inhibiting the swallowing and respiration process, and may cause death by suffocation. Piglets develop the pulmonary form of this disease through the inhalation of infected dust. The characteristic signs of pulmonary form anthrax is lobar pneumonia and exudative pleurisy. When pharyngitis occurs, the bloodstained froths may be present in the mouth [9,64]. In chronic gastrointestinal tract involvement, the clinical sign may be vomiting, icterus, enteritis, diarrhea, and constipation [4]. Some infected swines may recover after a few days of illness, but others develop fatal bacteremia.

## Diagnosis

When an animal has died from anthrax, it is better to avoid handling the animal, sample collection, and processing because of the risk of human infection, bacterial sporulation, and environmental contamination.

### Clinical pathology

**Figure 2.**
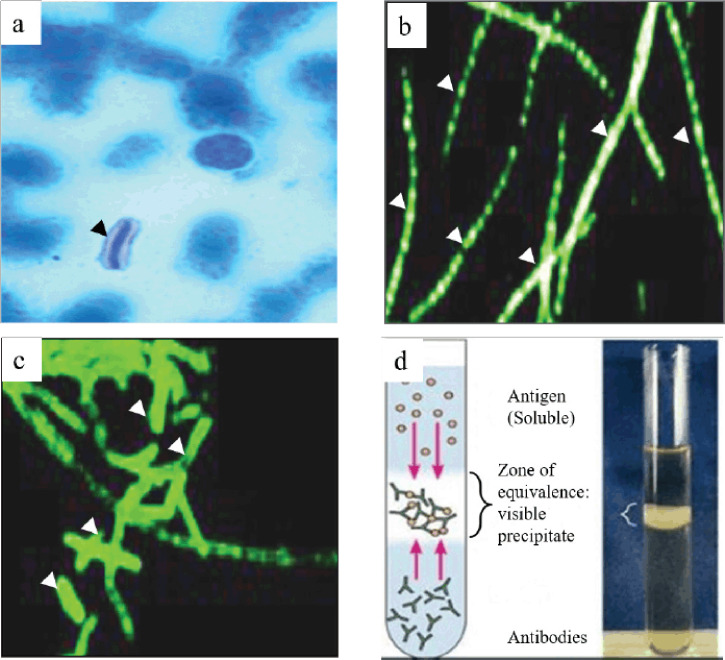
Staining and immunological techniques are used for the diagnosis of anthrax. (a) M’Fadyean staining, (b) FAT using monoclonal antibodies for cell wall, (c) and capsule were useful for the detection and identification of anthrax bacilli. In advanced decomposition, detection of the bacteria in the carcass is almost impossible where a precipitation test (Ascoli’s thermo precipitation test) is suitable for diagnosing anthrax (d). Black and white arrowheads indicate the bacteria after staining. Figures a–d were taken from or kindly provided by Anne Boyer (Anthrax Toxin Team Leader, CDC), De et al. [69], and Microbe Notes [83], respectively.

The hematology and blood chemistry examinations are not recommended due to the possibility of zoonotic risk. If undertaken, it should take the maximum level of protection. The *B. anthracis* may be identified in a stained smear from the live animals by Gram-stain or polychrome methylene blue (M’Fadyean stain) stain. The Gram-stained smear showed Gram-positive, thick, long, straight bacilli with square or truncated ends with parallel sides, usually arranged in single pairs or chains of three or four bacilli. The M’Fadyean stained smear showed the capsulated bacilli (pink capsule surrounding dark blue bacilli, often square-ended, singly, or in short chains) under the microscope (Fig. 2a) [20]. The capsule was stained pink in poly-D-glutamic acid staining, surrounding purple-stained vegetative bacilli [65].

In the early stage of infection, a few numbers of bacilli may be present in the bloodstream. In this case, the blood culture or collected blood injected into the guinea pig is satisfactory [9]. The blood culture is considered the gold standard method; however, it takes between 12 h and 5 days to detect *B. anthracis* [66–68].

The fluorescent antibody technique (FAT) and monoclonal antibodies are helpful for the identification of bacilli. The FAT is used to examine blood smears and tissue sections, and the monoclonal antibodies help for specific identification of anthrax organisms (Fig. 2b and c) [9,69]. Additionally, the Ascoli’s thermo precipitation test (Fig. 2d) and the polymerase chain reaction (PCR) are used for the antigen identification from the tissues of dead animals and environmental samples, respectively [9,20]. The Ascoli’s thermo precipitation test is not strictly specific for *B. anthracis*, and is not suitable for this bacteria identification in the specimen from the environment. On the contrary, PCR is useful for confirming the identity and virulence of the bacilli isolates [70].

The bacilli diagnosis by smear is more difficult when the animal has decomposed after death, and the vegetative forms of bacilli die. In this case, the immunochromatographic test for antigen has shown better results, which can detect PA from serum, blood, or other body fluids due to the present high concentration of PA in these samples, and this test has high specificity. It does not provide a positive test result in a recently vaccinated animal. The immunochromatographic test for detecting anti-protective antigen (anti-PA) antibodies may be valuable for the retrospective diagnosis confirmation [9,71,72]. In some cases, when the antibiotic treatment is applied, the animal passage may be necessary because the organism identification from blood smear or culture may be difficult. The antibody developed at the late stage of the disease; at that time, most probably, the bacilli were absent in the blood. For that reason, the serological test, like enzyme-linked immune sorbent assay will be used for retrospective studies [9,20].

When sending infected materials and conducting laboratory studies, strict biosecurity should be followed. For instance, it is necessary to consult with the local authority and the diagnostic laboratory before planning to ship the sample. The biosafety level (BSL) 2 laboratory is needed for the diagnostic test. But the BSL3 laboratory should be employed when the work involves raising the organism, the possibility for aerosol formation, or activities with antibiotic-resistant strains. If one wants to handle and process an anthrax-suspected sample, the author request that readers (he/she) should further read the guidelines of the World Health Organization (WHO) for the transport of an infectious substance and anthrax and the microbiological laboratory, and operational safety [73,74].

### Necropsy findings

Necropsy should not be performed on suspected anthrax cases because the spores will be formed at the site of exposed animals and possibly expose anthrax to humans. The presence of incomplete or absent rigor mortis with dark tarry blood oozing from the mouth, nostrils, anus, and marked bloating indicated the disease [64]. If the carcass is opened unintentionally, common necropsies observed include blood being dark thickened and failing to clot, hemorrhages on the serosal surface of the abdomen, thorax, epicardium, endocardium, and gastrointestinal tract mucosa and enlarged, dark red, or black, soft, semifluid spleen. When the skull is open, meningitis may observe. The liver, kidneys, and lymph nodes are congested and extended. In horses and pigs, the enlargement of the local lymph nodes and the subcutaneous swellings containing gelatinous material are common in this disease [9].

If an animal (especially ruminants) suddenly dies without previous symptoms, it should lead to a suspicion of anthrax. When anthrax is suspected as the cause of death, the infected carcass should not be opened as it initiates sporulation, contaminates the environment, and poses a health hazard to personnel and other adjacent animals. The diagnosis can be confirmed by aseptically obtaining a postmortem blood sample from a peripheral vein (e.g., the jugular vein or ear vein) and taking extraordinary measures like using double-layer air-tight leakproof containers during transportation of samples to the laboratory. Veterinarians and others should avoid skin contact with possibly contaminated carcasses and soil. Personal protective equipment (PPE) should be worn, such as impermeable gloves, boots, and clothes. After a confirmed anthrax diagnosis, the affected property should be quarantined, the possibly exposed stock should be vaccinated, dead animals should be safely disposed of by burning or other reliable procedures, and polluted sites should be disinfected [75]. The author requests the reader follow the WHO guidelines for transporting infectious substances for further knowledge [74].

### Differential diagnosis

In farm animals, the differential diagnosis includes many causes of sudden death and many causes of a blood clotting disorder.

#### In cattle and sheep

The clostridial infections, lightning strikes, and bloat must be consider in the cattle and sheep. In cattle, some other diseases also are considered. They are anaplasmosis, bacillary hemoglobinuria, acute leptospirosis, acute poisonings by lead, sweet clover, and bracken fern [64].

#### In horse

In horses, acute infectious anemia, clostridiosis, African horse sickness, colic, purpura, lead poisoning, ionophore toxicity, acute selenium toxicity, snakebite, sunstroke and lightning strike, third-degree heart block, malignant hyperthermia, and anaphylactic drug reaction must be considered before a final diagnosis [64].

#### In pigs

Acute classical swine fever, African swine fever, and malignant pharyngeal edema are considered in swine [64].

## Treatment

Anthrax bacterium remains susceptible to many antibiotics. The “susceptible antibiotics” for anthrax bacilli are penicillin, oxytetracycline, amoxicillin, chloramphenicol, ciprofloxacin, doxycycline, erythromycin, gentamicin, and sulfonamides. However, only penicillin and oxytetracycline are the most fruitful in the field [76]. The fever is present in the early stages of the disease before the other signs are noticeable. During that time, the animal responds well to proper treatment with penicillin. The commonly recommended doses of penicillin G sodium/potassium are 20,000 international units/kilogram (IU/kg), 12 hourly, intravenous (IV), and procaine penicillin is 22,000 IU/kg intramuscular (I/M), 12 hourly for the first 2 days. After 2 days, penicillin G sodium/potassium is 22,000 IU/kg, 24 hourly, IV, and procaine penicillin is 44,000 IU/kg, I/M, 24 hourly, following 3 days [9]. The drug oxytetracycline given 10 mg/kg daily at IM or IV is effective. In the initial period of therapy, the daily dose should be divided and given for 12 hours. The penicillin drug combined with streptomycin is also curative [20]. The different types of penicillin drugs need to follow the different administration routes, such as the penicillin G procaine injection is prohibited from being used intravenously because this may cause severe or life-threatening side effects or death.

Also, the hyperimmune anthrax serum has been suggested with antibiotic therapy. However, the antiserum is not usually produced or used in any place to treat anthrax in animals [20,76]. Penicillin, together with streptomycin, is regarded as the choice of treatment for animals showing clinical symptoms of anthrax. However, a few countries do not allow antibiotic treatment rather mandating slauther and proper disposal of animals affected with anthrax [20].

## Control

The anthrax control may be carried out by interrupting the infection source, adequately monitoring the disease, discarding the anthrax carcass properly, correctly disposing of the contaminated material, properly disinfecting the infected area, and correctly immunizing the susceptible animals [20].

When an outbreak occurs from a defined infection source, early steps should be to break the infection cycle, including discontinuation of the infection’s origin. For example, if the infection enters by feed items, immediately contaminated feed items and infection source should be appropriately destroyed. The infected farm should be quarantined and prevent the introduction of new animals into the infected area. When vectors like flies can be suspected of spreading this disease, flies should be adequately controlled [20].

The epidemiological data of this organism’s characteristics, its eco-epidemiological niche, and the risk factor determination of the natural occurrence played a principal role in anthrax spot identification. The early diagnosis and reporting of this disease outbreak help proper preparation and prompt response to reduce the further spread to animals and prevent human infection [31].

The infected carcasses should not be opened, it should be burned and discharged as early as possible. Another way of infected carcass disposal is by deep burial (6 feet), and previously, quicklime (calcium oxide) was recommended as an anthrax disinfectant for infected carcass covered with soil mixed with quicklime (3:1). However, current research claims that exposing anthrax spores to calcium may help them survive and thrive. For this reason, using lime or any calcium products for anthrax carcass disposal is not recommended. It is less desirable due to the risk of future spread of spore by disturbances of soil layer during plowing, soil erosion, digging by scavenging animals, and the water contamination that results in a new outbreak of this disease [31,77,78].

The least desirable option for disposal of anthrax carcasses is for the infected animal to keep intact, not moved, and prevent the access of scavenging animals and humans, resulting in natural decomposition. The vegetative bacteria cannot form spores in a carcass. But, it is inactivated by the putrefaction process. There is a possibility of environmental contamination by the bloody discharges from the natural orifices of the dying animals, but this reduces by scorching the affected site after complete putrefaction [31].

When the carcass and infected material are disposed of delay, it is better to apply 5% formaldehyde on the infected carcass and contaminated surrounding area and cover it with double-thickness plastics. The formalin-treated carcass is left *in situ* for some days before disposal. The formalin would kill anthrax organisms shed by the dead animal and preserve the skin, maintaining the anaerobic environment inside the carcass. On the contrary, the natural putrefaction processes in the carcass would kill the vegetative anthrax bacterium. However, the formalin-treated carcass does not discourage the scavengers or flies. It is better to segregate all infected animals properly, and the farm should keep them quarantined [9,20].

Disinfection of contaminated floor or room, fertilizer, meat, hides, wool, etc., needs special attention. The vegetative form of bacteria is eliminated by regular disinfectants or warmth (60°C for a few minutes) when applied shortly after the animal has died. It is sufficient for the contaminated room, abattoir, and necropsy room. When spore formation starts within a few hours of exposure to air, it is very difficult or almost impossible to disinfectant in an ordinary way. In those cases, a strong disinfectant like 5% Lysol for at least 2 days, formalin, or sodium hydroxide (5 to 10%) is helpful. The appropriate amounts (8 L/m^2^) of peracetic acid (3% solution) in the soil are effective sterilant against anthrax spore. The contaminated shoes are disinfected by placing them in a plastic bag and later introducing ethylene oxide. It is better to sterilize the hides and wool by gamma irradiation. The contaminating clothes fix with 10% formaldehyde. The contaminated area must be disinfected if the human skin is contaminated [9].

The most effective method of controlling anthrax is vaccination. The veterinary anthrax vaccines are classified into two categories. Currently, the most used and effective vaccines in the world are live attenuated, toxigenic, non-capsulating (pXO1+/pXO2–), *B. anthracis* strain Sterne 34F2, acquired from a virulent bovine isolate in the 1930s [79–81]. Another form of anthrax vaccine is the Pasteur-type vaccine (pXO1-/2+), which has some drawbacks, such as varying susceptibility to vaccines in different animal species, a ≥3% mortality risk, and is currently rejected by a majority of countries worldwide. The Sterne vaccine administered subcutaneously (s/c) annually: 1 ml for large ruminants and 0.5 ml for small ruminants. The anthrax vaccination should be administered 2–4 weeks before the disease outbreak, and a second dose is commonly needed 2–4 weeks after the first dose. In general, immunity develops after 7 days of vaccination [76].

When anthrax cases are found for the first time in a certain locations, the contact animals should be treated with either hyperimmune serum or vaccinated. Vaccination failure infrequently occurs, which is primarily due to poor vaccination coverage. So, to ensure maximum vaccination coverage, a door-to-door strategy could be adopted [82]. Ordinarily, the vaccinated cow milk is discarded for 72 h, and animals are retained from slaughter for 45 days after vaccination. However, after vaccination, the bacilli of the Sterne vaccine are not found in the milk and blood for 10 and 7 days, respectively [9].

## Conclusion

Anthrax is quite susceptible to antibiotic therapy, and the clinical course is often so quick that infected animals may not be able to be treated. So, focusing on control strategies for anthrax-suspected areas is essential. The anthrax control strategies recommended include immunization, quarantine, and proper handling and disposal of the carcass. In addition to control strategies, implementing appropriate hygienic procedures at slaughterhouses and dairy industries will ensure the safety of products of animal origin considered for human consumption. The detailed knowledge obtained from this review would help decision-makers in the livestock sector to improve the technical capacity of regional and national veterinary services.

## List of Abbreviations

USA, United States of America; gm+, Gram-positive WHO, World Health Organization; PA, Protective antigen; EF, Edema factor; LF, Lethal factor;TNF, Tumor necrosis factor; IL-1, Interleukin-1; ET, Edema toxin; LT, Lethal toxin; PCR, Polymerase chain reaction;BSL, Biosafety level; IU, International unit; kg, Kilogram; IV, Intravenous; I/M, Intramuscular; mg, Milligram;s/c, Subcutaneous; ml, Milliliter
